# Cardiovascular risk factors across job roles and work shifts in a Brazilian Military Police cohort: a cross-sectional study

**DOI:** 10.3389/fpubh.2025.1716547

**Published:** 2025-12-17

**Authors:** Daniel Franceschini Palmieri, Leonardo Borges Ferreira, Iúri Leão de Almeida, Vinicius C. Fiusa, Fiorella Jamilé Bazán Gonzales, João Pedro de Oliveira Bicalho Santos, Ana Cláudia Cavalcante Nogueira, Luiz Sérgio Fernandes de Carvalho, Alexandre Anderson de Sousa Munhoz Soares

**Affiliations:** 1 Medical Sciences Postgraduate Program, Universidade de Brasilia (UnB), Brasilia, Brazil; 2 Department of Health Assistance, Federal District Military Police, Brasilia, Brazil; 3 Higher Institute of Police Sciences, Federal District Military Police, Brasilia, Brazil; 4 Faculty of Social, Work and Organizational Psychology, University of Brasília, Brasilia, Brazil; 5 Data Lab for Quality of Care and Outcomes Research (LaDaQCOR), Universidade Catolica de Brasilia (UCB), Brasilia, Brazil; 6 Aramari Apo Institute for Advanced Health Education and Research, Brasilia, Brazil

**Keywords:** cardiovascular risk factors, police officers, occupational health, shift work, lawenforcement, metabolic syndrome, hypertension, obesity

## Abstract

**Background:**

Police officers are recognized as a high-risk group for cardiovascular disease (CVD), but it remains unclear how specific occupational factors, such as job roles and demanding shift schedules, modulate this risk. This study aimed to assess the prevalence and clustering of cardiovascular risk factors in a cohort of Brazilian military police officers and to investigate their associations with job type and work schedule.

**Methods:**

A cross-sectional analysis involving 436 male military police officers in Brazil was performed. Prevalence of cardiovascular risk factors and overall cardiovascular health scores were determined based on six key metrics: elevated blood pressure, dyslipidemia, dysglycemia, obesity, smoking status, and physical activity levels. Health profiles and risk factor prevalence were compared across administrative versus operational roles and different work shifts using Pearson’s chi-squared tests for categorical data and non-parametric tests for non-normally distributed continuous data.

**Results:**

The population (median age 46.0 years) exhibited a high burden of risk factors, with over 80% being overweight or obese and 95% presenting with elevated blood pressure or hypertension. Consequently, nearly 90% of the officers were classified as having moderate or high cardiovascular risk, while only 3% met ideal health metrics. This high-risk profile was uniformly distributed across the force, with no statistically significant differences observed between job types or work schedules.

**Conclusion:**

This cohort of military police officers exhibits a severe and systemic burden of cardiovascular risk factors, pervasive throughout the force regardless of specific job roles or shift schedules. Given this widespread risk profile, the findings strongly recommend systemic, force-wide institutional health promotion initiatives, rather than programs targeted only at specific diseases, roles, or shifts.

## 1 Introduction

Atherosclerosis-related cardiovascular diseases (CVD), such as coronary artery disease (CAD) and cerebrovascular disease, are the leading cause of morbidity and mortality worldwide (1). The impact of these conditions is particularly severe in Brazil, where they account for approximately 30% of all deaths, of which 61% are attributed specifically to CAD and cerebrovascular disease (2). A significant portion of these devastating conditions are preventable. Estimates suggest that up to 90% of myocardial infarctions and 70% of all CVD are directly linked to modifiable cardio-metabolic and behavioral risk factors (3).

Police populations exhibit a higher prevalence of cardiovascular risk factors and CVD compared to civilian populations, military firefighters, civilian public servants, and armed forces personnel (4–8). This is a documented global phenomenon. A meta-analysis estimated the worldwide prevalence of metabolic syndrome (MS) in police officers at 26.2%, significantly exceeding the 8.3% found in general military personnel (9). This risk is also supported by cohort studies showing police have a higher incidence of acute myocardial infarction (6), alongside high rates of MS (32.7%) (10) and hypertension (40.4%) (11) compared to other populations.

The Brazilian scenario appears particularly severe. Local studies confirm a high burden of MS, with prevalence rates for hypertension reaching as high as 55.76% (12). Furthermore, a high prevalence of overweight or obesity is consistently reported across Brazilian police forces, affecting 70 to 83% of officers (7, 13, 14). This is often coupled with high rates of physical inactivity (44–73%) (13, 15) and some form dyslipidemia (45–64%) (14).

Factors present in police work, such as chronic stress, irregular hours, and intense physical demands, are strongly associated with an increased risk of metabolic syndrome and CVD (16, 17). However, it is less clear how this risk is distributed across different roles and work schedules within the police force, with most studies lacking the detailed clinical and laboratory data needed to compare, for example, operational versus administrative personnel. Early identification of those factors is crucial for implementing effective preventive strategies, including lifestyle interventions, stress management programs, and systematic medical follow-up (15, 18–20).

Cardiovascular risk stratification can be performed through various methods, ranging from individual factor assessment with binary cut-off points, to multivariate statistical models such as the Framingham Risk Score and the American Heart Association (AHA)’s Pooled Cohort Equations (21, 22). An intermediately complex but still valuable approach is the aggregation of factors, such as in Life’s Simple 7, also proposed by AHA, which evaluates seven modifiable health factors (physical exercise, diet, body weight, smoking, cholesterol, blood pressure, and blood glucose) (23).

With approximately 405,000 active members, the Brazilian Military Police represent a substantial and high-risk occupational group (24). Given the uncertainty regarding the internal distribution of cardiometabolic risk within this population, a deeper understanding of these factors is crucial. Therefore, this study aimed to determine the prevalence and clustering of major cardiovascular risk factors and examine their associations with job role and work shift among members of a Brazilian Military Police cohort.

## 2 Methods

### 2.1 Study design and population

This study is a cross-sectional analysis of baseline data from a prospective cohort designed to assess the relationship between cardiovascular risk factors, inflammatory markers, psychological factors, and carotid atheromatosis with cardiovascular events in military police officers from the metropolitan area of Brasília, Brazil. Participants were recruited via an open invitation disseminated through internal institutional channels. Clinical evaluations were scheduled using a digital platform, resulting in a voluntary convenience sample of active-duty officers who met the inclusion criteria.

The required sample size was calculated using EpiInfo (version 7.2.5.0), based on an estimated population of 6,600 active-duty male officers aged >40 years. The calculation assumed a conservative 50% prevalence for key risk factors to ensure adequate statistical power for comparing subgroups within this cross-sectional analysis, considering potential variability and ensuring robust estimates.

Between September 2021 and April 2024, 456 volunteers were assessed at the cardiology outpatient clinic of the police medical center. Inclusion criteria were: male sex, age over 40 years, active-duty status, and no known atherosclerotic disease (myocardial infarction, unstable angina, percutaneous coronary intervention, coronary artery bypass surgery, stroke, or peripheral vascular disease). After excluding 10 individuals for statin use (a pre-specified exclusion criterion) and 10 for missing functional data, the final study cohort comprised 436 participants.

Comparisons of health profiles and risk factors were made based on work assignment (administrative vs. operational) and the following work schedules: 6 h/day, 5 days/week; 12/36 h (meaning 12 h on duty followed by 36 h off duty); 12/60 h; and 24/72 h.

### 2.2 Data collection and clinical definitions

Data were collected by a board-certified cardiologist during outpatient visits and managed using the REDCap platform. The collected data included epidemiological information, job characteristics, medical history, physical examination findings, and laboratory parameters.

Laboratory tests, including complete blood count, fasting blood glucose, glycated hemoglobin (HbA1c), lipid profile, and high-sensitivity C-reactive protein (hs-CRP), were performed by the Sabin laboratory network. Participants were instructed to postpone testing if they were taking anti-inflammatory drugs or within 14 days of an acute inflammatory condition.

For this study, variables were defined as follows:

- Physical activity: Classified as sedentary (none), insufficient (<150 min/week), or active (≥150 min/week).
- Blood pressure (BP): Classified according to recent guidelines (25) as non-elevated (SBP < 120 mmHg and DBP < 70 mmHg), elevated (SBP 120–139 mmHg or DBP 70–89 mmHg), or hypertension (SBP ≥ 140 mmHg, DBP ≥ 90 mmHg, or use of antihypertensive medication).
- Glucose metabolism: Classified based on American Diabetes Association criteria (26) as normal (fasting glucose <100 mg/dL and HbA1c < 5.7%), prediabetes (glucose 100–125 mg/dL or HbA1c 5.7–6.4%), or diabetes (glucose ≥126 mg/dL, HbA1c ≥ 6.5%, or use of antidiabetic medication).
- Dyslipidemia: Diagnosed based on Brazilian guidelines (27) in individuals with low-density lipoprotein cholesterol (LDL-C) ≥ 130 mg/dL, high-density lipoprotein cholesterol (HDL-C) ≤ 40 mg/dL, and/or triglycerides ≥150 mg/dL.

### 2.3 Cardiovascular health score

Participants were categorized into cardiovascular risk groups based on a health score adapted from the American Heart Association’s Life’s Simple 7 (17). The dietary metric was excluded as these data were not collected due to technical and logistical constraints within the study’s protocol. The health score was calculated by summing the number of ideal metrics met from the following six parameters: (1) BMI < 25 kg/m^2^; (2) sufficient physical activity (≥150 min/week); (3) non-elevated blood pressure (SBP < 120 mmHg and DBP < 80 mmHg); (4) healthy glucose metabolism (fasting glucose < 100 mg/dL and HbA1c < 5.7%); (5) healthy lipid profile (LDL-C < 130 mg/dL, HDL-C > 40 mg/dL, and triglycerides < 150 mg/dL); and (6) no history of smoking. Based on this score, participants were classified as low risk (5–6 metrics), moderate risk (3–4 metrics), or high risk (0–2 metrics).

### 2.4 Statistical analysis

Data were analyzed using RStudio (version 4.4.2). Categorical variables were compared using Pearson’s chi-squared test. Continuous variables were assessed for normality using the Shapiro–Wilk test, and as all were non-normally distributed, comparisons were performed using the Mann–Whitney U test for two groups or the Kruskal–Wallis H test for multiple groups. Missing data for continuous variables were imputed using the Multivariate Imputation by Chained Equations (MICE) package with predictive mean matching (m = 5, maxit = 50). A two-tailed *p*-value < 0.05 was set as statistically significant.

## 3 Results

### 3.1 Sociodemographic and job characteristics

The study cohort consisted of 436 male military police officers with a median age of 46.0 years. Officers in administrative roles were more likely to have a postgraduate degree (22.8% vs. 8.8%, *p* < 0.001) and had a longer median service time (24.0 vs. 23.0 years, *p* = 0.007) compared to those in operational roles. As expected, higher military ranks were predominantly found in the administrative group (*p* < 0.001). Further demographic and occupational details are presented in Table 1.

**Table 1:** Epidemiology and job characteristics, total population and stratified by type of work (administrative or operational).

Variable	Total (*n* = 436)	Adm. (*n* = 197)	Ope. (*n* = 239)	*p*-value
Age (years), Mdn[IQR]	46.0 [43.0–49.0]	46.5 [43.0–49.0]	46.0 [43.0–48.0]	0.145†
Skin color, *n* (%)				0.043*
Mixed	279 (64.0)	123 (62.4)	156 (65.3)	
White	117 (26.8)	62 (30.3)	55 (23.1)	
Others	40 (9.2)	11 (5.9)	28 (12.0)	
Education, n (%)				<0.001*
High school	14 (3.2)	6 (3.2)	8 (3.4)	
Bachelor’s	355 (81.4)	145 (73.6)	210 (87.9)	
Postgraduate	66 (15.1)	45 (22.8)	21 (8.8)	
Marital status, *n* (%)				0.271*
Single	33 (7.6)	12 (5.4)	21 (9.0)	
Married/cohabiting	376 (86.2)	176 (90.3)	200 (83.3)	
Divorced	24 (5.5)	7 (3.8)	17 (7.3)	
Other	3 (0.7)	2 (0.5)	1 (0.4)	
Service (years), Mdn [IQR]	24.0 [20.0–26.0]	24.0 [21.0–27.0]	23.0 [19.0–25.0]	0.007†
Military rank, *n* (%)				<0.001*
Corporal	8 (1.8)	0 (0.0)	8 (3.3)	
Sergeant	344 (78.9)	135 (68.5)	209 (87.4)	
Warrant Officer	36 (8.3)	21 (10.7)	15 (6.3)	
Lieutenant	9 (2.1)	7 (3.8)	2 (0.8)	
Captain	11 (2.5)	5 (2.7)	5 (2.1)	
Major,	17 (3.9)	17 (8.6)	0 (0.0)	
Lieu.-Col./Col.	10 (2.3)	10 (5.1)	0 (0.0)	
Monthly overtime shifts, *n* (%)				0.478*
None	169 (39.4)	73 (37.1)	96 (40.2)	
1–3	47 (11.2)	22 (11.2)	25 (10.5)	
4–6	193 (45.6)	83 (42.1)	110 (46.0)	
>6	16 (3.8)	10 (5.1)	6 (2.5)	

*Chi-square test. †Mann–Whitney test. Adm: administrative IQR: interquartile range. Mdn: median. Ope: operational.

### 3.2 Clinical profile and high prevalence of risk factors

A high burden of cardiometabolic risk factors was observed across the cohort (Table 2). The majority of officers (81.1%) were overweight (55.0%) or obese (26.1%), with a median BMI of 27.7 kg/m^2^ and a median abdominal circumference of 98.0 cm. While most participants were physically active (58.3%) and never-smokers (87.6%), clinical parameters indicated significant risk.

**Table 2:** Clinical characteristics, total population and stratified by type of work (administrative or operational).

Variable	Total (*n* = 436)	Adm. (*n* = 197)	Ope. (*n* = 239)	*p*-value
BMI (kg/m^2^), Mdn [IQR]	27.7 [25.4–30.1]	27.7 [25.4–30.1]	27.7 [25.4–30.1]	0.897†
Abd. circ. (cm), Mdn [IQR]	98.0 [91.0–105.0]	99.0 [92.0–106.0]	97.0 [91.0–105.0]	0.122†
BMI classification, *n* (%)				0.371*
Normal weight	82 (18.8%)	42 (21.3%)	40 (16.7%)	
Overweight	240 (55.0)	102 (51.8)	138 (57.1)	
Obese	114 (26.1%)	53 (26.9%)	61 (25.5%)	
Physical exercise, *n* (%)				0.416*
Sedentary	118 (27.1)	58 (29.4)	60 (25.1)	
Insufficient	64 (14.7)	31 (15.7)	33 (13.8)	
Active	254 (58.3)	108 (54.8)	146 (61.1)	
Smoking history *n* (%)				0.563*
Never smoked	382 (87.6)	169 (85.8)	213 (89.1)	
Ex-smoker	34 (7.8)	18 (9.1)	16 (6.7)	
Current smoker,	20 (4.6)	10 (5.1)	10 (4.2)	
SBP (mmHg), Mdn [IQR]	131.0 [122.0–139.0]	131.0 [121.0–139.0]	131.0 [122.0–139.0]	0.780†
DBP (mmHg), Mdn [IQR]	84.0 [77.0–91.0]	84.0 [77.0–91.0]	83.0 [76.0–91.0]	0.294†
Blood pressure categories, *n* (%)				0.013*
Non-elevated	20 (4.6)	3 (1.5)	17 (7.1)	
Elevated	270 (61.9)	129 (65.5)	141 (59.0)	
Hypertension	146 (33.5)	65 (33.0)	81 (33.9)	
Blood glucose (mg/dL), Mdn [IQR]	90.0 [85.0–96.0]	90.0 [85.0–96.0]	89.0 [85.0–95.0]	0.549†
HbA1c (%), Mdn [IQR]	5.5 [5.3–5.8]	5.5 [5.3–5.8]	5.5 [5.3–5.8]	0.869†
Glucose metabolism, *n* (%)				0.819*
Normal	244 (56.0)	107 (54.3)	137 (57.3)	
Pre-diabetes	164 (37.6)	77 (39.1)	87 (36.4)	
Diabetes	28 (6.4)	13 (6.6)	15 (6.3)	
Tot. Chol. (mg/dL), Mdn [IQR]	206.6 [184.8–229.9]	204.8 [186.6–224.8]	208.0 [184.4–234.3]	0.517†
HDL-C (mg/dL), Mdn [IQR]	42.0 [37.0–47.0]	42.0 [37.0–47.0]	42.0 [37.0–48.0]	0.672†
LDL-C (mg/dL), Mdn [IQR]	132.0 [113.0–150.0]	131.0 [114.0–148.0]	134.0 [113.0–152.5]	0.213†
Non-HDL-C, Mdn [IQR]	156.0 [137.8–179.0]	155.0 [138.0–177.0]	157.0 [137.5–180.0]	0.553†
Trig. (mg/dL), Mdn [IQR]	140.0 [98.8–186.0]	143.0 [98.0–188.0]	138.0 [99.5–180.5]	0.582†
Dyslipidemia, *n* (%)	370 (84.9%)	167 (84.8%)	203 (84.9%)	1*

*Chi-square test; †Mann–Whitney test; Adm, administrative; BMI, body mass index; DBP, diastolic blood pressure; HbA1c, glycated hemoglobin; HDL-C, high-density lipoprotein cholesterol; IQR, interquartile range; LDL-C, low-density lipoprotein cholesterol; Mdn, median; Ope, operational; SBP, systolic blood pressure; Trig, triglycerides.

An overwhelming 95.4% of the cohort presented with either elevated blood pressure or hypertension, with a median SBP of 131.0 mmHg and DBP of 84.0 mmHg. Notably, this burden was not evenly distributed across job roles. The administrative group had a significantly lower proportion of officers with non-elevated blood pressure compared to the operational group (1.5% vs. 7.1%; *p* = 0.013), highlighting a greater prevalence of hypertension among non-operational staff (Table 2). Regarding glucose metabolism, 44.0% of officers had prediabetes (37.6%) or diabetes (6.4%). Dyslipidemia was also highly prevalent, affecting 84.9% of the participants.

### 3.3 Cardiovascular health score profile

**Figure 1:**
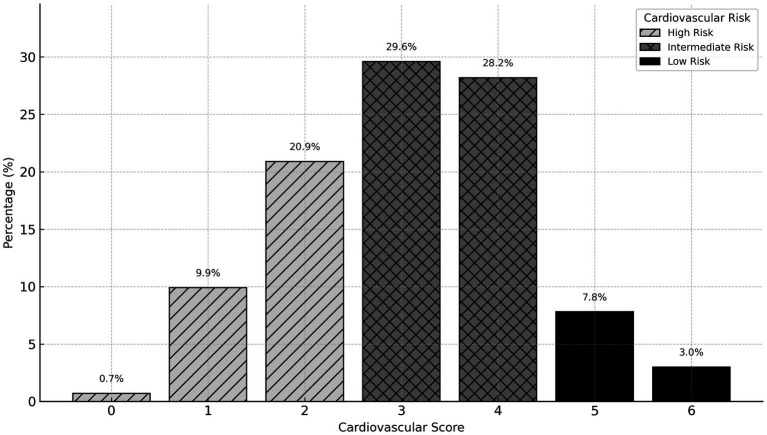
Histogram of cardiovascular health score.

**Figure 2:**
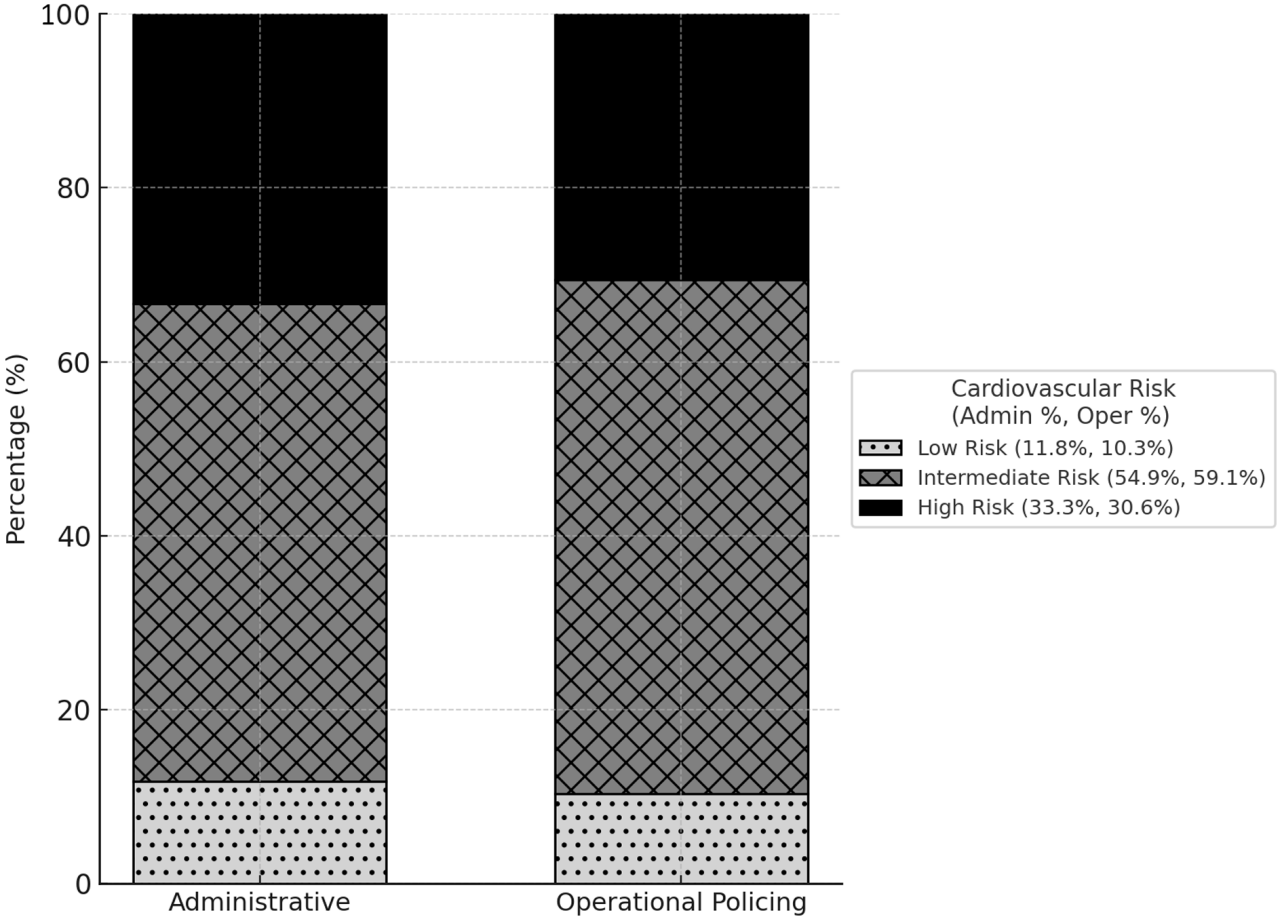
Cardiovascular health score distribution stratified by type of work (administrative or operational).

The overall cardiovascular health of the cohort was poor, as measured by a six-metric health score. The distribution of scores was negatively skewed, with a high concentration of officers having unfavorable profiles (Figure 1). Only 10.8% of the sample met at least five of the six ideal cardiovascular health metrics. Consequently, most officers were classified as having moderate (57.8%) or high (31.4%) cardiovascular risk. This distribution was consistent between administrative and operational officers, with no significant difference observed between the groups (Figure 2, *p* = 0.628).

### 3.4 Analysis by work schedule

**Figure 3:**
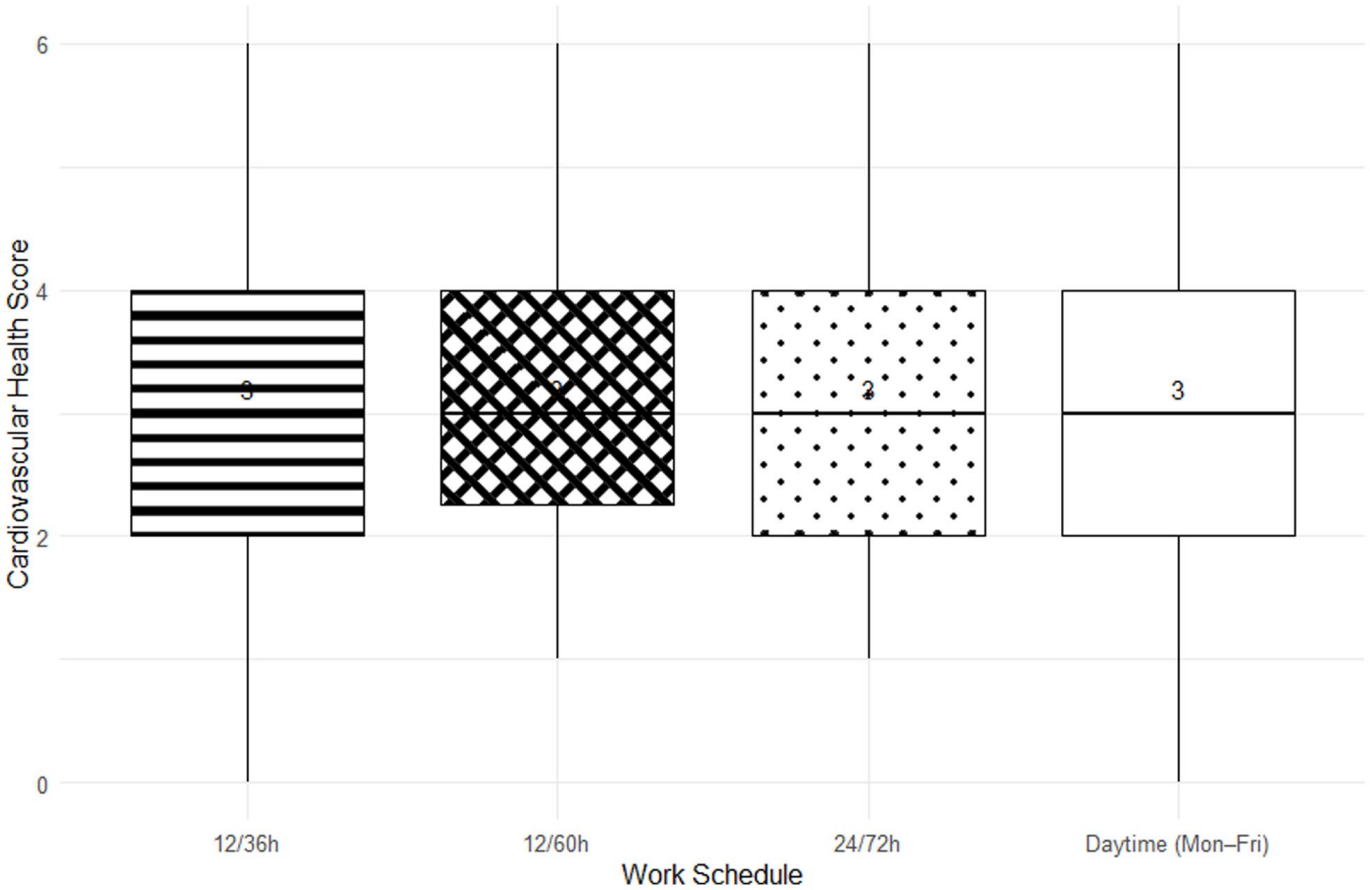
Cardiovascular health score distribution stratified by work schedule (6 h/day 5x/week, 12/36 h, 12/60 h, or 24/72 h).

When the cohort was stratified by four different work schedules, the overall cardiovascular risk profile remained consistent across the groups. No statistically significant differences were found in key anthropometric, hemodynamic, or laboratory parameters (Table 3). However, a non-significant trend was observed: officers working the 12/36 h shift showed numerically higher values for BMI, systolic blood pressure, and LDL-C, alongside a higher prevalence of insufficient physical activity, suggesting a potentially less favorable risk profile. The distribution of cardiovascular health scores was also similar across all schedule types (Figure 3).

**Table 3:** Clinical characteristics (Mdn and proportions) stratified by work schedule (6 h/day 5x/week, 12/36 h, 12/60 h, or 24/72 h).

Variable	6 h/day 5x/week (*n* = 185)	12/36 h (*n* = 77)	12/60 h (*n* = 102)	24/72 h (*n* = 72)	*p*-value
BMI (kg/m^2^),	27.8 [25.6–30.2]	28.4 [25.9–30.8]	27.3 [25.2–29.8]	27 [25.4–28.8]	0.237†
Abd. circ. (cm), Mdn [IQR]	99 [92–106]	96 [91–102]	99 [92.2–105]	97.5 [91–105.5]	0.174†
SBP (mmHg), Mdn [IQR]	131 [123–140]	132 [120–140]	129 [121.2–136.8]	131 [122.8–137.8]	0.559†
DBP (mmHg), Mdn [IQR]	85 [78–91]	85 [76–93]	80 [76–88]	84.5 [75–91]	0.103†
Tot. Chol. (mg/dL), Mdn[IQR]	204.6 [183.2–224.4]	213.6 [190–238]	203.9 [178.9–228.2]	208.3 [190.6–234.1]	0.191†
HDL-C (mg/dL), Mdn [IQR]	42 [37–47]	41 [37–47]	41.5 [36–47]	44 [40–50.2]	0.183†
LDL-C (mg/dL), Mdn [IQR]	132 [112–147]	140 [119–159]	126 [109–151.5]	136.5 [118.8–153]	0.0871†
Non-HDL-C, Mdn [IQR]	155 [134–175]	169 [141–191]	156 [133–175.5]	154.5 [140.5–181.5]	0.194†
Trig. (mg/dL), Mdn [IQR]	140 [95–188]	151 [96–195]	135.5 [100.2–181]	133.5 [102.2–169.5]	0.463†
Elevated BP or HTN, *n* (%)	98.4% (182)	92.2% (71)	93.1% (95)	94.4% (68)	0.0756*
Prediabetes or DM, n (%)	44.9% (83)	48.1% (37)	42.2% (43)	40.3% (29)	0.774*
Overweight or obesity, *n* (%)	80% (148)	81.8% (63)	81.4% (83)	83.3% (60)	0.938*
Current smoker, *n* (%)	4.3% (8)	2.6% (2)	6.9% (7)	4.2% (3)	0.588*
Insufficient PA, *n* (%)	43.2% (80)	51.9% (40)	36.3% (37)	34.7% (25)	0.103*
High TG or Low HDL, *n* (%)	64.3% (119)	63.6% (49)	60.8% (62)	54.2% (39)	0.489*
High LDL-C, *n* (%)	61.1% (113)	74% (57)	54.9% (56)	66.7% (48)	0.0557*

*Chi-square test; †Kruskal-Wallis test; BMI, body mass index; DBP, diastolic blood pressure; DM, diabetes mellitus; HTN, hypertension; IQR, interquartile range; LDL-C, low-density lipoprotein cholesterol; Mdn, median; PA, physical activity; SBP, systolic blood pressure; Trig, triglycerides.

## 4 Discussion

This study revealed a remarkably high prevalence of cardiovascular risk factors among a Brazilian military police officers cohort, with nearly 90% exhibiting moderate to high-risk profiles based on the presence of hypertension, diabetes, dyslipidemia, obesity, smoking and/or sedentarism. This composite risk assessment, while more focused than the American Heart Association’s comprehensive Life’s Simple 7 framework, captures key biological determinants of cardiovascular health, with elevated BMI serving as a validated proxy for poor dietary patterns when direct nutritional assessment is unavailable (28, 29). The prevalence observed in our police cohort starkly contrasts with Brazilian national data, where only 63.3% of adults achieve ideal biological health metrics (blood pressure, glucose, and cholesterol) and a mere 0.5% meet comprehensive cardiovascular health criteria (30). Most significantly, our systematic comparison between administrative and operational personnel revealed that this elevated risk burden was uniformly distributed across all job roles and shifts, despite administrative officers showing higher rates of elevated blood pressure and longer service tenure. This homogeneous risk distribution suggests that cardiovascular risk in law enforcement transcends specific duties and reflects systemic factors inherent to the policing profession.

### 4.1 Systemic risk, job roles, and lack of protection of administrative work

The homogeneity of cardiovascular risk across administrative and operational roles is a critical finding that challenges conventional assumptions about occupational health in law enforcement. This uniformity is particularly striking given that administrative officers presented a higher burden of elevated blood pressure and longer service tenure. We propose this paradox is explained by a compound risk profile in administrative personnel, where the apparent protection of a non-operational role is largely illusory. These officers face both the chronic risks associated with sedentary work and age, alongside periodic exposure to the acute stressors of policing.

Two primary factors explain this dynamic. First, the functional distinction between roles is blurred. The high prevalence of extra operational shifts among administrative officers (62% in our sample), combined with shared stressors like mandatory uniform use and open carry of firearms, creates a persistently stressful environment that parallels frontline duties (31–33). Second, a “reverse healthy worker effect” may be at play, where organizational processes systematically concentrate individuals with poorer clinical profiles—often due to longer careers—in less physically demanding administrative roles (34). This is supported by our finding of longer service tenure in this group, a factor independently associated with a nearly five-fold increase in 10-year cardiovascular disease risk in other police cohorts (35).

The higher blood pressure burden in the administrative group is thus explained by this convergence of factors: a baseline of older age and longer service duration linked to increased hypertension odds (36), compounded by prolonged sedentary work and the fatigue related to extra shifts (37–39). Consequently, when these officers with an elevated chronic risk are placed in high-stress operational situations during extra shifts (40), they form a uniquely vulnerable population for acute cardiovascular events.

Our findings of risk homogeneity are reinforced by a growing body of literature. Yates et al. (41) reported virtually identical hypertension prevalence rates between operational (60.5%) and non-operational (60.0%) police officers. Similarly, studies comparing police with civilian office workers found no significant differences in overall cardiovascular risk, suggesting that the systemic occupational environment—rather than specific job duties—is the primary determinant of health outcomes (38).

### 4.2 The influence of work schedules on cardiometabolic health

Although our analysis of work schedules did not yield statistically significant differences, a clinically meaningful pattern emerged. Officers working shifts with shorter recovery periods, particularly the 12/36-h schedule, consistently exhibited numerically less favorable cardiometabolic profiles. Conversely, schedules with longer rest intervals were associated with more favorable parameters across multiple biomarkers.

While not statistically significant, possibly due to sample size limitations, this consistent trend suggests a clinically relevant relationship between recovery time and metabolic health. This observation aligns with substantial evidence that circadian misalignment from inadequate rest impairs metabolic regulation, promotes atherosclerosis, and increases oxidative stress and insulin resistance (37, 42, 43). This trend supports the hypothesis that sufficient rest may be protective and warrants investigation in larger, longitudinal studies.

### 4.3 Career-long risk accumulation and comparative prevalence

The prevalence of specific risk factors in our cohort was alarmingly high, a finding best understood as the culmination of career-long risk accumulation. A recent U. S. cohort study found that officers’ cardiorespiratory fitness was below age-matched standards at all career stages, with the deficit widening significantly with age, while BMI and body fat progressively increased (44).

This trajectory is consistent with findings from Brazilian officers, where those over 40 already exhibit a higher prevalence of hypertension, diabetes, and obesity compared to younger counterparts (7). Our cohort, with a median age of 46 and 24 years of service, perfectly illustrates the clinical manifestation of this accelerated, age- and tenure-related risk accumulation. The outcomes are stark when compared to external benchmarks:

- Overweight and Obesity: The prevalence in our sample (81.1%) far exceeds the Brazilian male average of 57.5% (45) and reported rates among police officers in Canada (62.6%) (46) and Germany (55.0%) (10), though it aligns with other Brazilian police studies (70.8–83.9%) (47, 48). The median abdominal circumference (98.0 cm) also surpassed national cardiovascular risk cut-offs (49, 50). High BMI in military populations is a known driver of metabolic syndrome and is linked to increased cardiovascular mortality (51, 52).
- Hypertension: The observed prevalence of hypertension (33.5%) was higher than that reported in Brazilian civilians (28.7%) (53) and other Brazilian police units (21–28%) (14, 54). The fact that only 4.6% of officers had optimal blood pressure is particularly concerning.
- Glucose Metabolism: While the diabetes rate (6.4%) was similar to the national average (55), the prevalence of prediabetes (37.6%) was exceptionally high, far surpassing rates in the general adult population (7.5–18.5%) (56). This highlights a significant, and often undetected, future risk for type 2 diabetes, as progression from prediabetes to diabetes can reach 70% without intervention; however, structured lifestyle modifications have been proven to reduce this risk by more than 50% (57).
- Dyslipidemia: The prevalence of dyslipidemia (composite) (84.9%) was also exceptionally high, with values for high LDL-C, low HDL-C, and high triglycerides all exceeding national averages for men (58) and rates in other police cohorts (12, 59).

### 4.4 Proposed interventions

Controlling these modifiable risk factors is crucial. Integrated interventions that combine nutritional education, physical activity programs, and psychological support have been shown to reduce cardiovascular risk factors in workers exposed to chronic stress (60, 61). Fitness programs in law enforcement have successfully enhanced lipid profiles, VO₂max, and muscle strength (62–65), while stress management can reduce fatigue (66). Facilitating access to leisure-time physical activity through employer-sponsored programs, such as gym subsidies, represents a particularly high-impact strategy. Meta-analytic evidence shows that workplace wellness programs can yield a substantial return on investment, with medical cost savings of approximately $3.27 and absenteeism reduction benefits of $2.73 for every dollar invested (67). For law enforcement agencies, implementing incentivized health and wellness programs is a practical way to address the systemic cardiovascular risk observed in our cohort, accommodating the irregular schedules characteristic of policing. Such initiatives are directly linked to enhanced occupational performance and career longevity (68). To be most effective, these fitness programs should be embedded within multifactorial approaches to controlling hypertension, dyslipidemia, and diabetes (69, 70). This highly educated population—with over 80% holding a bachelor’s degree—offers a favorable setting for interventions focused on self-efficacy and health literacy, which are key mediators of positive health behaviors (71, 72). Such preventive initiatives not only improve clinical outcomes but also offer significant economic benefits by reducing long-term medical costs (73).

### 4.5 Strengths and limitations

The study’s primary strength is its use of a comprehensive set of clinical and laboratory data to assess cardiovascular health in a large, well-characterized cohort of police officers, a significant advance over studies relying on self-reported data. The detailed analysis of job roles and work schedules provides a nuanced view of occupational factors.

The main limitations are the study’s cross-sectional design, which precludes causal inference. The use of a voluntary convenience sample may introduce selection bias and limits the generalizability of our findings. While volunteer bias often trends toward healthier participants (the ‘healthy volunteer effect’), potentially meaning the true risk burden in the broader police population is even higher than observed here, the opposite bias is also possible. Officers with existing health concerns or greater awareness of cardiovascular risk might have been more motivated to participate, potentially inflating the observed prevalence rates (74). The precise direction and magnitude of this bias remain unknown, further emphasizing caution when generalizing these results. Furthermore, future research should consider analyzing work schedules grouped by recovery time rather than as distinct categories. Such an approach could increase statistical power and potentially reveal significant associations between rest duration and cardiometabolic health that were not detected in our stratified analysis. Additionally, data on key potential mediators such as diet, sleep patterns, and perceived stress were not collected, limiting a deeper analysis of the pathways to the observed high cardiovascular risk. These factors should be a priority for future longitudinal research.

## 5 Conclusion

This sample of military police officers exhibits a severe and systemic burden of cardiovascular risk factors that is pervasive throughout the force, irrespective of their specific job roles or shift schedules. This unfavorable health profile, which exceeds that of the general population, highlights an urgent need for comprehensive, institution-wide health promotion and prevention programs. Prioritizing the cardiovascular health of officers is not merely a matter of individual well-being but is a strategic imperative to sustain operational readiness, resilience, and their continued service to society.

## Data Availability

The datasets generated and analyzed during the current study are available from the corresponding author on reasonable request, subject to approval by the command of the Federal District Military Police. Requests to access the datasets should be directed to dfpalmieri@gmail.com.
